# Endoplasmic Reticulum–Mitochondria Contact Sites and Neurodegeneration

**DOI:** 10.3389/fcell.2020.00428

**Published:** 2020-06-18

**Authors:** Lingna Xu, Xi Wang, Chao Tong

**Affiliations:** ^1^Ministry of Education Key Laboratory of Biosystems Homeostasis and Protection and Innovation Center for Cell Signaling Network, Life Sciences Institute, Zhejiang University, Hangzhou, Zhejiang, China; ^2^The Second Affiliated Hospital, School of Medicine, Zhejiang University, Hangzhou, Zhejiang, China

**Keywords:** endoplasmic reticulum, mitochdonrion, contact sites, neurodegeneration, autophagy

## Abstract

Endoplasmic reticulum–mitochondria contact sites (ERMCSs) are dynamic contact regions with a distance of 10–30 nm between the endoplasmic reticulum and mitochondria. Endoplasmic reticulum–mitochondria contact sites regulate various biological processes, including lipid transfer, calcium homeostasis, autophagy, and mitochondrial dynamics. The dysfunction of ERMCS is closely associated with various neurodegenerative diseases, including Parkinson’s disease, Alzheimer’s disease, and amyotrophic lateral sclerosis. In this review, we will summarize the current knowledge of the components and organization of ERMCSs, the methods for monitoring ERMCSs, and the physiological functions of ERMCSs in different model systems. Additionally, we will emphasize the current understanding of the malfunction of ERMCSs and their potential roles in neurodegenerative diseases.

## Introduction

Endoplasmic reticulum (ER) forms interconnected networks of membrane tubules and sacs that play a major role in the synthesis, modification, and transport of proteins and lipids in the eukaryotic cells (Schwarz and Blower, 2016). Mitochondrion serves as a powerhouse and metabolic center for the production of ATP and precursors of macromolecules, such as proteins, lipids, DNA, and RNA (Devine and Kittler, 2018). The contact sites between ER and mitochondria are not only critical for the communications between these two important organelles but also serve as a platform to regulate other cellular events (Cohen et al., 2018). Since the early 1970s, ER–mitochondria contact sites (ERMCSs) have been observed through ultrastructural electron microscopy. The subcellular fractionation studies also indicated a close association between the membranes of ER and mitochondria. Although ERMCSs are conserved structures in all eukaryotic cells, the tethering molecules are diverse across species. Recent studies have developed many methods to monitor ERMCS and identified multiple tethering complexes that mediate ERMCS formation. Previous studies have extensively investigated the functions of ERMCS at the cellular levels. Several diseases, including neurogenerative disorders, are associated with abnormal ERMCSs (Paillusson et al., 2016). Since there are many high-quality reviews published recently, we will briefly give an introduction to the organization and functions of ERMCSs in this review (Phillips and Voeltz, 2016; Lackner, 2019; Moltedo et al., 2019).

## The Organization of ERMCSs

**FIGURE 1:**
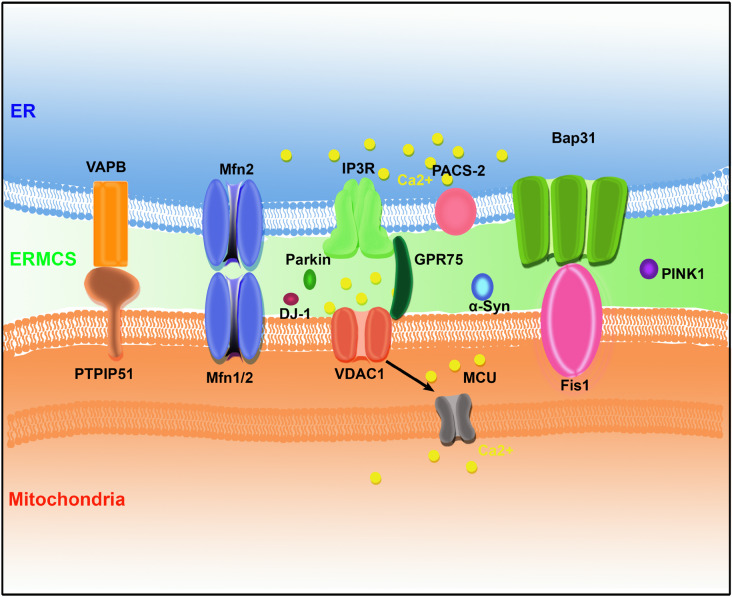
The protein organization at ER–mitochondria contact sites (ERMCSs) in mammalian systems. Several pairs of proteins located on mitochondria and ER surface to form tethers, including vesicle-associated membrane protein (VAMP)-associated protein (VAP) B (VAPB)–protein tyrosine phosphatase-interacting protein-51 (PTPIP51), Mfn1/2, inositol 1,4,5-trisphosphate receptor (IP3R)–glucose-regulated protein 75 (GPR75)–voltage-dependent anion channel (VDAC1) complex, and B-cell receptor-associated protein 31 (Bap31) (Bap31)–fission 1 homolog (Fis1). Neurodegenerative disease-related proteins such as α-synuclein (α-Syn), DJ-1, PINK1, and Parkin were concentrated at ERMCSs.

In the last few decades, several pairs of protein complexes have been identified that bridge the ER and mitochondria. The mitochondria and ER membrane components mediate the formation of ERMCSs with the help of some cytosolic proteins to promote the exchange of cellular components and signals between the ER and mitochondria (Figure 1).

### The Tethering Molecules in Yeast

ER–mitochondria encounter structure (ERMCS), a protein complex in yeast, was reported to mediate the contacts between ER and mitochondria. The mitochondrial outer membrane proteins (Mdm10 and Mdm34), cytosolic protein (Mdm12), and integral ER protein (Mmm1) mediate the tethering between mitochondria and ER to form ERMES. Gem1, the yeast ortholog of Miro, is enriched in the contact sites and regulates ERMES (Kornmann et al., 2011). Additionally, the ER membrane protein complex (EMC) interacts with the mitochondrial translocase of outer membrane (TOM) complex to tether ER to the mitochondria (Lahiri et al., 2014). In contrast to ERMES, EMC is conserved in higher organisms. The role of mammalian EMC in tethering ER to the mitochondria is not known. Ltc1/Lam6 is also reported to regulate the contact between the ER and mitochondria in yeast (Elbaz-Alon et al., 2015; Murley et al., 2015). Ltc1/Lam6 is conserved among the mammalian cells. However, the role of mammalian orthologs of Ltc1/Lam6 in ERMCSs is not known.

### The Tethering Molecules in Mammals

#### Mitofusin 2

In mammals, several tether molecules are reported to mediate the formation of ERMCSs. Mitofusin 2 (MFN2) is a dynamin-like GTPase that is located not only on the mitochondrial outer membrane but also is enriched in the interface between ER and mitochondria. MFN2 can form a homotypic dimer and a heterotypic dimer with its paralog MFN1, which plays a major role in the mitochondrial outer membrane fusion. In 2008, MFN2 was reported to tether ER to the mitochondria and to regulate mitochondrial calcium uptake from ER. *Mfn2* ablation in the mouse embryonic fibroblasts (MEFs) and HeLa cells increases the distance between ER and mitochondria and decreases the calcium transfer from ER to the mitochondria (De Brito and Scorrano, 2008). Several groups have reported new components that regulate ER–mitochondrial tethering via MFN2 (Cerqua et al., 2010; Sugiura et al., 2013; Daniele et al., 2014; Morales et al., 2014). However, later studies have challenged the role of MFN2 in mediating the formation of ERMCS. The loss of *Mfn2* does not decrease the formation of ERMCS but increases both ERMCS formation and calcium trafficking between the ER and mitochondria (Cosson et al., 2012; Filadi et al., 2016; Leal et al., 2016). This indicated that MFN2 serves as an antagonist to ERMCS formation. Further studies are needed to elucidate the role of MFN2 in tethering ER to the mitochondria.

#### VAPB and PTPIP51

Vesicle-associated membrane protein (VAMP)-associated proteins (VAPs) are integral ER membrane proteins that contain an N-terminal major sperm protein (MSP) domain, a central coiled-coil region, and a C-terminal transmembrane domain. The vertebrates have two VAPs (VAPA and VAPB). Mutations in VAPB cause rare forms of spinal muscular atrophy (SMA) and amyotrophic lateral sclerosis 8 (ALS8) in patients. VAPs play an important role in membrane trafficking, lipid transfer and metabolism, unfolded protein response (UPR), and autophagy (Kaiser et al., 2005; Lev et al., 2008; Zhao et al., 2018). VAPs interact with various proteins containing the FFAT-motif, which comprises consensus EFFDAXE amino acid sequence and serves as a versatile access point for the ER (Murphy and Levine, 2016). Several FFAT-motif-containing proteins mediate the contact between ER and other organelles. Protein tyrosine phosphatase-interacting protein-51 (PTPIP51) is a mitochondrial outer membrane protein containing an FFAT-motif that binds to VAPB (De Vos et al., 2012). Overexpression of either VAPB or PTPIP51 increases the ERMCS formation and calcium transfer from ER to mitochondria. The RNA interference (RNAi)-mediated silencing of VAPB or PTPIP51 or overexpression of ALS mutant form of VAPB (VAPBP56S) decreases the ERMCS formation and disturbs the calcium exchange between these two organelles (Stoica et al., 2014). TDP-43 and FUS are two proteins that are pathologically linked to ALS and frontotemporal dementia (FTD). TDP-34 and FUS modulate the interaction between VAPB and PTPIP51 via the activation of GSK-3β protein kinase and therefore perturbs ERMCS (Stoica et al., 2014; Stoica et al., 2016). The Parkinson’s disease (PD)-related protein, α-synuclein, binds to VAPB and disrupts the interaction between VAPB and PTPIP51, which leads to the loss of contact between ER and mitochondria (Paillusson et al., 2017).

#### B-Cell Receptor-Associated Protein 31 and Its Binding Partners

B-cell receptor-associated protein 31 (Bap31) is an integral ER membrane protein containing an N-terminal membrane-bound region with three predicted transmembrane helices and a cytosolic C-terminal domain with one or two predicted coiled coils (Iwasawa et al., 2011). During apoptosis, the mitochondrial fission protein, fission 1 homolog (Fis1) interacts with Bap31 to bridge the ER and mitochondria and promotes the caspase-8-mediated cleavage of Bap31 into the pro-apoptotic p20Bap31 (Chandra et al., 2004). The ablation of phosphofurin acidic cluster sorting protein 2 (PACS-2), a multifunctional ER-associated vesicular sorting protein, leads to Bap31-dependent mitochondrial fragmentation and uncoupling of the ER from the mitochondria (Simmen et al., 2005). However, further studies are needed to confirm whether PACS-2 functions as a component of the Fis1–Bap31 complex or as a regulator of Fis1–Bap31 interaction.

Bap31 also interacts with Bcl-2, which is localized in the mitochondria. The interaction between Bap31 and Bcl-2 is facilitated by the interaction between CDIP1 and Bap31 in the ER during ER-stress (Namba et al., 2013).

A recent study reported that Bap31 could also form an ER–mitochondrial bridging complex by interacting with translocase of the outer mitochondrial membrane 40 (Tom40). The Bap31–Tom40 complex facilitates nuclear-encoded mitochondrial protein translocation and mitochondrial homeostasis (Namba, 2019).

#### VDAC1–Grp75–IP3R

The voltage-dependent anion channel (VDAC), which is located at the mitochondrial outer membrane, is a key component that mediates calcium transport to the mitochondria (Rapizzi et al., 2002). Voltage-dependent anion channel physically interacts with inositol 1,4,5-trisphosphate receptor (IP3R), the ER calcium release channel, through the molecular chaperone glucose-regulated protein 75 (Grp75). These tripartite complexes colocalize on the mitochondrial-associated membranes (MAMs) and directly enhance the mitochondrial calcium uptake (Szabadkai et al., 2006). This suggested that the VDAC1–Grp75–IP3R complex may serve as an ER–mitochondria tether. The silencing of Grp75 abolishes the mitochondrial calcium uptake. Transglutaminase type 2 (TG2) interacts with Grp75 in the MAMs. Disrupting the TG2–Grp75 interaction decreases the number of ER–mitochondria contact sites (D’eletto et al., 2018). Sigma-1 receptor (Sig-1R) binds to IP3R at MAM to regulate ER–mitochondrial interorganellar calcium signaling (Hayashi and Su, 2007). Other modulators, such as mitochondrial calcium uniporter (MCU; Xu et al., 2018) and etoposide-induced protein 2.4 (EI24; Yuan et al., 2019), were also reported to regulate the VDAC1–Grp75–IP3R scaffolds. However, IP3R ablation does not affect ER–mitochondria contacts, which indicates that VDAC1–Grp75–IP3R scaffold is not involved in physical tethering (Csordas et al., 2006).

## The Functions of ERMCSs

**FIGURE 2:**
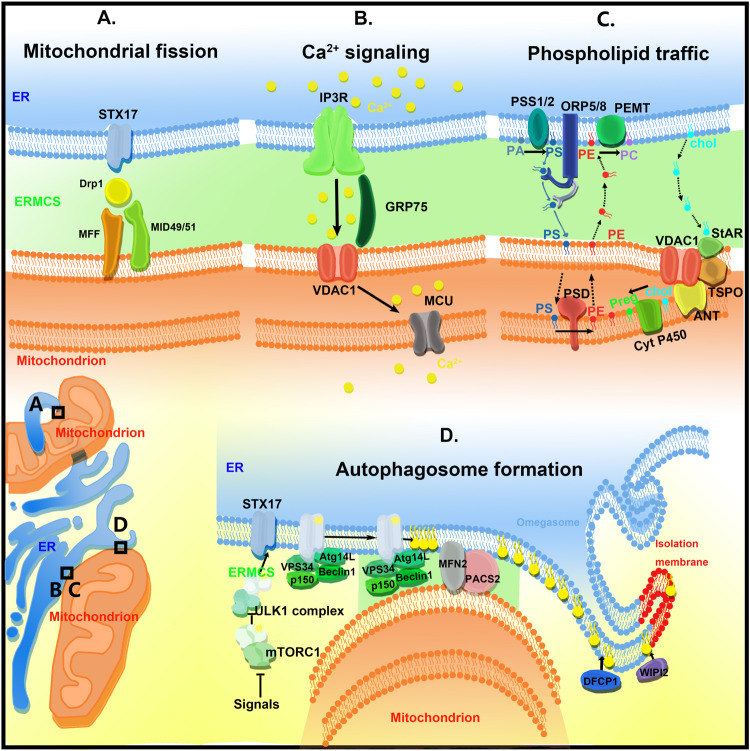
The functions of ER–mitochondria contact sites (ERMCSs). The typical ERMCSs are illustrated at the bottom left corner. The boxed regions are enlarged to show the detailed cellular events and molecular organizations. **(A)** Mitochondria fission occurs at the ERMCSs. ER-located protein Syntaxin 17 (STX17) presents at ERMCS and mediates mitochondrial division by determining the localization and activity of Drp1. **(B)** The VDAC1–Grp75–IP3R tripartite complex serves as the major platform that coordinates calcium transfer between ER and mitochondria. Mitochondrial inner membrane located mitochondrial calcium uniporter (MCU) takes up the calcium transported from VDAC1. **(C)** The ERMCSs form the platform for phospholipid traffic between ER and mitochondria. **(D)** The pre-autophagosome/autophagosome proteins re-localize to the ERMCS to promote the initiation of autophagosome formation.

Endoplasmic reticulum–mitochondria contact sites regulate a variety of biological processes, including calcium homeostasis, lipid transfer, autophagy, and mitochondrial dynamics (Figure 2).

### Regulation of Mitochondrial Dynamics

Mitochondria are highly dynamic organelles that undergo fusion and fission to maintain their healthy state. Interestingly, the ER tubules define the position of mitochondrial fission sites (Friedman et al., 2011). In both yeast and mammalian cells, mitochondrial division initially occurs at the ER-mitochondrial contact sites, and the ER–mitochondrial contacts are formed before the recruitment of fission machinery proteins, such as Drp1 and Mff (Friedman et al., 2011). Inverted formin 2 (INF2), an ER-located protein, regulates actin polymerization and drives the mitochondrial constriction and division in the mammalian cells (Korobova et al., 2013). Syntaxin 17 is located on the tubular, smooth ER membranes. Syntaxin 17 is involved in the ER–Golgi intracellular trafficking and autophagy. Recently, Syntaxin 17 was reported to be present at the ER–mitochondrial contact sites and to mediate mitochondrial division by determining the localization and activity of Drp1 (Arasaki et al., 2015). Sept2, a subset of proteins localized to the mitochondrial constrictions, was reported to physically bind to Drp1 and to mediate Drp1-dependent mitochondrial division in the mammalian cells (Pagliuso et al., 2016). The contact sites not only mark the mitochondrial fission sites but also mark the replication sites of mtDNA (Lewis et al., 2016). The mtDNA is localized in the matrix. Thus, it would be interesting to determine the communication between ERMCS and mtDNA replication machinery in the mitochondrial matrix. The results of high-resolution microscopy imaging analysis indicated that some mitochondrial fusion events also occur at the ERMCSs (Guo et al., 2018). However, the molecular mechanisms underlying this process are still elusive.

### Calcium Transfer

ER lumen is the major calcium store in the mammalian cells. The ER calcium level is determined by sarco-ER Ca^2+^ transport ATPases (SERCAs), intraluminal calcium-binding proteins, such as calreticulin, calnexin (CNX), and BiP/GRP78. SERCA2b is a ubiquitously expressed isoform of SERCAs that is critical for ER calcium uptake. Calnexin and thioredoxin-related transmembrane protein 1 (TMX1) regulate SERCA2b activity. Calnexin is enriched at the ER luminal side of the ERMCSs through palmitoylation and interaction with PACS-2. Transmembrane protein 1 belongs to the family of protein disulfide isomerases. Transmembrane protein 1 has a single transmembrane domain and is palmitoylated at the cytosolic stretch, which is required for its targeting to the ERMCS (Lynes et al., 2012). Transmembrane protein 1 inhibits the activity of SERCA2b, which can be antagonized by CNX, the positive regulator of SERCA2b. The loss of TMX1 increases the ER calcium store.

Upon stimulation, calcium is released to the cytosol through IP3R on the ER membrane. The external signals stimulate the production of Ins(1,4,5)P3 that binds and activates IP3R and triggers the calcium release from ER to the cytosol and enhances the local calcium concentration. The calcium flux from ER to mitochondria is critical for multiple mitochondrial functions. Basal calcium oscillation in mitochondria is required for multiple metabolic processes (Gellerich et al., 2010). The calcium overload in the mitochondria leads to the opening of the mitochondrial permeability transition pore and subsequentially results in cell death (Baumgartner et al., 2009). As the MCU complex has low calcium affinity, ER and mitochondria must be in close proximity for producing high local calcium concentration. Thus, ERMCS is a hot spot for calcium transfer between the ER and mitochondria.

IP3R, which is enriched in the ERMCS, interacts with VDAC1 located at the mitochondrial outer membrane via a chaperone, Grp75. The resulting VDAC1–Grp75–IP3R complex serves as the major platform and chaperone complexes that coordinate calcium transfer between ER and mitochondria (Bononi et al., 2012). MCU localized at the mitochondrial inner membrane takes up the calcium transported from VDAC1. When ER calcium level is low, Sig-1R is released from BIP/GPR78 to stabilize IP3R3 at ERMCSs and promote prolonged ER calcium release (Hayashi and Su, 2007; Figure 2B).

### Lipid Synthesis and Exchange

The cellular transport and homeostasis are dependent on the extensive transport of lipids and their precursors between ER and mitochondria (Osman et al., 2011; Figure 2C). Endoplasmic reticulum is a major membrane lipid synthesis center in the cells. The mitochondrial membrane has high levels of phospholipid and low levels of sterols and sphingolipids. Sphingolipids and phospholipids, such as phosphatidic acid (PA), phosphatidylserine (PS), phosphotidylcholine (PC), and phosphatidylinositol (PI), are synthesized in the ER. Cardiolipin (CL), a diglycerophospholipid, is a mitochondrial-specific phospholipid enriched in the inner mitochondrial membrane. Cardiolipin is synthesized in the mitochondria at the matrix side of the inner membrane using PA transported from the ER (Tatsuta et al., 2014). Phosphatidylserine is converted to phosphatidylethanolamine (PE) by PS decarboxylase (PSD) on the inner mitochondrial membrane. The mitochondrion-derived PE is transferred back to the ER, where it serves as a precursor for synthesizing PC. As mitochondria are not integrated into the classical vesicular trafficking routes, non-vesicular mediated transports that occur at the contact sites of membranes play major roles in the lipid transport between the ER and mitochondria. Several key enzymes mediate phospholipid synthesis, such as phosphatidylserine synthase-1/2 (PSS1/2) and phosphatidylethanolamine N-methyltransferase 2 (PEMT2), an enzyme implicated in PC synthesis. These enzymes are localized to the ERMCSs (Cui et al., 1993). In yeast, ERMES-deficient mitochondria exhibit an altered membrane lipid composition and an impaired conversion of PS to PC (Kornmann et al., 2009). However, there is no direct evidence to show that ERMES participates in the lipid transfer directly. The EMC complex in yeast is also involved in PS shuttling from ER to mitochondria (Lahiri et al., 2014). However, the EMC proteins do not have a lipid-binding domain and, therefore, may regulate lipid transfer indirectly. In mammals, further studies are needed to elucidate the mechanism underlying lipid transfer at ERMESs. Oxysterol-binding protein (OSBP)-related protein 5 and 8 (ORP5/ORP8) were recently reported to be localized at the ERMCSs and to potentially mediate the transport of phospholipid (probably PS) between two organelles (Galmes et al., 2016).

In addition to phospholipid, key regulators of triacylglycerol synthesis and steroidogenesis, such as acyl-CoA/diacylglycerol acyltransferase 2 (DGAT2; Stone et al., 2009) and steroidogenic acute regulatory protein (StAR; Prasad et al., 2015) are enriched in the ERMCSs. Long-chain-fatty-acid-CoA ligase 4 (FACL4; Lewin et al., 2001), an enzyme that mediates the ligation of fatty acids to coenzyme A (CoA), and acyl-coenzyme A:cholesterol acyltransferase-1 (ACAT1/SOAT1), an enzyme that catalyzes the generation of cholesterol esters, is also enriched in ERMCSs (Lewin et al., 2002). These findings indicate that the ERMCSs are closely related to lipid exchange between the ER and mitochondria, and lipid metabolism.

### Regulation of Autophagy Process

Autophagy is an intracellular bulk degradation process, which is tightly regulated. Autophagy dysfunction is closely associated with numerous human diseases (Mizushima and Komatsu, 2011). The initiation of autophagy involves the formation of isolation membranes, which engulf some cytosolic components and damaged organelles. The isolation membranes are sealed to form double-membraned autophagosomes, which fuse with the lysosomes where the contents are degraded. Although the ER, mitochondria, and plasma membrane are reported to contribute to the original autophagosome membrane, the origin of the autophagosomal membranes is still controversial. In 2013, Hamasaki et al. (2013) demonstrated that ERMCSs mediate autophagosome formation. Upon starvation, the pre-autophagosome/autophagosome markers, ATG14 and ATG6, re-localize to the ERMCS to initiate autophagosome formation. The disruption of the ERMCSs decreases the number of ATG14-positive autophagosomes. Meanwhile, Syntaxin 17 is redistributed to ERMCSs to mediate PI3–kinase complex recruitment by physically binding to ATG14. PTPIP51 is also reported to bind to VAPB and to regulate autophagy (Gomez-Suaga et al., 2017). Increasing ERMCS formation by overexpressing VAPB or PTPIP51 impairs autophagosome formation. Conversely, decreasing the expression of VAPB or PTPIP51 stimulates autophagy. These data indicate that ERMCS is a hot spot to regulate the autophagy process (Figure 2D).

## ERMCSs and Neurodegeneration

**FIGURE 3:**
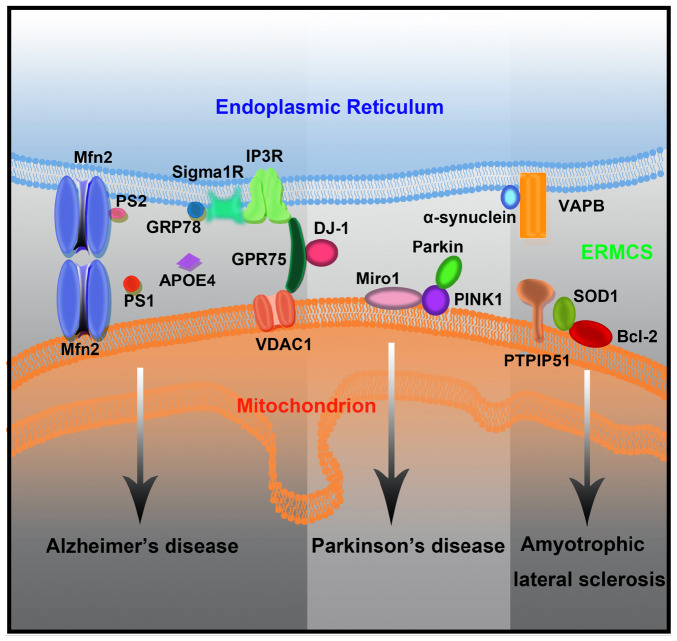
The mutant forms of neurodegenerative disease-associated protein lead to ERMCS defects and finally neuronal death.

ERMCSs have attracted widespread attention in neurodegenerative diseases, mainly because they are widely involved in processes closely related to mitochondrial function and cell survival. Several neurodegenerative disease-associated proteins are enriched in the ER–mitochondrial interface. Several studies have demonstrated that ERMCSs, which are the key regulators of lipid metabolism, Ca^2+^ homeostasis, and autophagosome formation, are affected by pathogenic mutations (Figure 3).

### Alzheimer’s Disease

Familial cases of Alzheimer’s disease (AD) are rare and are caused due to the mutations in the amyloid precursor protein (APP) or presenilins (PS1 and PS2). The APP Swe/Lon mouse model exhibit enhanced ER–mitochondria connection and enhanced mitochondrial calcium concentrations without marked changes in the lipid composition (Hedskog et al., 2013).

PS1 and PS2 are components of the γ-secretase complex, which is involved in APPβ processing (Naj and Schellenberg, 2017). Both PS1 and PS2 are enriched in the mitochondria-associated ER membranes (Area-Gomez et al., 2009). The mutations in PS2 increase ERMCS formation and enhance cholesteryl ester and phospholipid synthesis in the cellular models (Zampese et al., 2011; Area-Gomez et al., 2012). Additionally, PS2 mutations increase the ER–mitochondria connection in the fibroblasts of patients with sporadic AD (Area-Gomez et al., 2012). Although mutations in PS1 and PS2 can enhance cholesteryl ester and phospholipid synthesis, only PS2 can modulate the Ca^2+^ shuttling between ER and mitochondria (Zampese et al., 2011).

Sig-1R forms a Ca^2+^-sensitive chaperone complex with Bip/GRP78 and promotes Ca^2+^ release from the ER by stabilizing IP3R3. It has been proposed that Sig-1R forms part of the endogenous defense system against AD (Maurice and Goguadze, 2017). Reportedly, compounds with Sig-1R agonist activity possess neuroprotective abilities. These findings indicate that the activity of Sig-1R may prevent AD pathology, therefore, presenting a promising therapeutic target for AD (Jia et al., 2019).

Individuals carrying the ε4 allele of apolipoprotein E (ApoE4) have increased risk for developing AD compared to the ones carrying ApoE3, the most common isoform. Cells treated with ApoE4 containing astrocyte conditioned media (ACM) have increased synthesis of phospholipids and of cholesteryl esters compared to those treated with ApoE3 containing ACM, suggesting an upregulated ERMCS functions in the cells treated with ApoE4 containing ACM (Tambini et al., 2016).

Although most evidence illustrates that enhanced ERMCS activity is associated with AD, conflicting results have been suggested in other studies. It has been demonstrated that expressing a linker that can force contacts between mitochondria and the ER suppressed motor impairment and extended the lifespan in a *Drosophila* model of AD (Garrido-Maraver et al., 2020).

### Parkinson’s Disease

Parkinson’s disease is a progressive movement disorder with selective loss of dopaminergic (DA) neurons in the substantia nigra and accumulation of Lewy bodies consisting of α-synuclein aggregates in the patient’s brains. Most PD cases are sporadic, and less than 10% are familial cases caused by genetic mutations in genes, such as *SNCA*, *LRRK2*, *VPS35*, *PINK1*, *PARK2*, and *PARK7* (Liu et al., 2019).

α-Synuclein, a presynaptic protein encoded by *SNCA*, is detected in the ERMCSs. In HeLa cells, PD-associated synuclein mutations decreased ER–mitochondria connections, as well as phospholipid synthesis (Guardia-Laguarta et al., 2014). α-Synuclein binds to the ERMCS tether protein VAPB. The VAPB–PTPIP51 tethers were disrupted when either the wild type or the PD-associated mutant forms of α-synuclein were overexpressed. A similar disruption of the VAPB–PTPIP51 interaction was also observed in neurons derived from induced pluripotent stem cells from familial PD patients with an affected *SNCA* gene. Hence, the contacts between the ER and mitochondria were loosened, and Ca^2+^ signaling at neuronal ERMCSs was affected (Paillusson et al., 2017).

DJ-1 is a conserved multifunctional protein encoded by *PARK7*. Loss of DJ-1 leads to early-onset recessive familial PD. DJ-1 is enriched in ERMCSs and interacts with the VDAC1–Grp75–IP3R complex (Liu et al., 2019). In HeLa cells, overexpression of DJ-1 enhanced ER–mitochondrial calcium transfer and marginally increased ERMCSs (Ottolini et al., 2013). DJ-1 ablation disrupted the formation of the VDAC1–Grp75–IP3R complex, causing IP3R accumulation at ERMCSs. Furthermore, similar ERMCS defects were observed in the brain of DJ-1 knockout mice and sporadic PD patients. In *DJ-1* knockout M17 cell lines, the length of ERMCSs was greatly reduced, and the ER–mitochondria calcium transfer was significantly decreased (Liu et al., 2019).

*PARK2* is an important gene whose mutation is responsible for 50% of familial autosomal recessive PD cases and probably some sporadic PD cases (Wang et al., 2010). *PARK2* encodes an E3 ligase Parkin that play critical roles in mitophagy. Calì et al. demonstrated that the upregulation of *PARK2* can enhance the mitochondrial Ca^2+^ uptake from ER (Cali et al., 2013). Consistent with this study, Basso et al. observed that the tether between the ER and mitochondria decreased in *PARK2* mutant human fibroblasts. Furthermore, the locomotor deficit in the *Drosophila* model of PD can be rescued by expressing an ER–mitochondria synthetic linker to increase ER–mitochondria tethering (Basso et al., 2018). However, Gautier et al. revealed that the ER and mitochondria are closely associated and the ER–mitochondria Ca^2+^ transfer was enhanced in the primary cells from patients with PD presenting *PARK2* mutations (Gautier et al., 2016). The rationale underlying the differences in these findings remain unclear.

PINK1 is a protein kinase necessary for the recruitment of Parkin to the damaged mitochondria during mitophagy. In DA neurons with *PINK1* mutations, ERMCSs were strengthened, and the mitochondrial Ca^2+^ level was increased. Miro, a protein well known for axonal transport of mitochondria, mediated the effects of PINK1 on mitochondrial calcium and morphology. Miro overexpression mimicked *PINK1* mutant-induced mitochondrial Ca^2+^ elevation, which could be rescued by inhibiting the genes involved in calcium transfer in ERMCSs. Inhibition of Miro or components of ERMCSs rescued the defects induced by the *PINK1* mutation. Surprisingly, the Miro-mediated calcium transfer was independent of its mitochondrial transport activity (Lee et al., 2018). Recently, *RHOT1*, the gene coding for Miro1, was found to carry mutations in patients with PD. The structure and number of ERMCSs were altered, and Ca^2+^ homeostasis was impaired in patient-derived fibroblasts carrying *RHOT1* mutants (Berenguer-Escuder et al., 2019).

### Amyotrophic Lateral Sclerosis

Amyotrophic lateral sclerosis is a fatal late-stage neurodegenerative disorder, characterized by progressive loss of motor neurons, muscle weakness, spasms, and death within a few years of diagnosis. Over 90% of ALS cases occur sporadically, and familial cases of ALS are rare. Furthermore, approximately 15% of ALS present with frontotemporal lobar dementia (ALS/FTD). Familial ALS is caused due to mutations in the genes encoding for the antioxidant protein superoxide dismutase 1 (SOD1), the TAR-DNA binding protein 43 (TDP43), ubiquilin 2 (Ubqln2), and VAPB (De Mario et al., 2017; Perrone et al., 2020).

Dominant mutations in the gene encoding SOD1 leads to familial ALS and accumulation of SOD1 mutant proteins in the ERMCSs (Bruijn et al., 2004). Reportedly, the mutant SOD1 interacts with Bcl-2, affecting calcium homeostasis (Eckenrode et al., 2010).

Accumulation of TDP-43 is a hallmark of ALS/FTD pathology. Overexpression of wild type, as well as mutant TDP-43, leads to reduced ER–mitochondria association, decreased VAPB–PTPIP51 interaction, and affects Ca^2+^ homeostasis (Stoica et al., 2014).

In the spinal cord of patients with ALS, VAPB mRNA levels were decreased when compared to control subjects (Anagnostou et al., 2010). The P56S mutant form of VAPB has been associated with ALS type 8 (Perrone et al., 2020). VAPB–P56S has demonstrated altered affinity to PTPIP51 and increased Ca^2+^ release from ER stores, consequently elevating Ca^2+^ uptake by the mitochondria (De Vos et al., 2012).

## Tools for Studying ERMCSs

Since Copeland and Dalton first discovered ERMCSs in the pseudobranch glands of *Fundulus heteroclitus* by electron microscopy, various methods have been used to study the structure and function of ERMCSs. The function and structure of ERMCSs were gradually revealed after the establishment of protocols for isolating membrane contacts and high-resolution microscopy (Table 1).

**TABLE 1:** Advantages and disadvantages of the various experimental approaches to study endoplasmic reticulum–mitochondria contact sites (ERMCSs).

Approaches		Advantages	Disadvantages
**Study of ERMCS morphology and structure**			
Electron microscopy	Transmission electron microscopy (TEM)	• Golden standard	• Provides static, high-resolution ultrastructure information	• Suitable for samples with a large amount of contact sites	• Fixation may introduce artifacts
	Electron tomography (ET)	• Three-dimensional view of the subcellular structures	• Technically challenging	• “missing wedge” artifacts
	Scanning electron microscopy (SEM)	• Provides high-resolution 3D image	• Overcome the “missing wedge” artifacts	• Needs powerful computer to process large datasets
Epifluorescence and confocal microscopy	Super-resolution microscopy	• Suitable for both static and live-cell imaging	• Enable the high-resolution observation of ERMCS dynamics	• Optical diffraction limit	• Fixation may introduce artifacts
	FRET-based reporter	• Provides temporal quantitative measurements of contact distance	• Prolonged drug treatments can introduce artifacts
	Split green fluorescent protein (GFP)	• Different probes could be used to examine the narrow and wide contacts	• Less responsive to the subtle changes in the contacts
	Light-inducible ER–mitochondria tethering (LIT) system	• Temporally regulate the contacts	• Avoid side effects caused by continuous ER–mitochondria tethering	• Needs careful control
Split Rluc8		• Easy technique	• Needs careful control
Proximity ligation assay (PLA)		• Mainly used to detect the proximity between the two proteins	• Requires antibodies to the proteins of interest
**Detection of resident proteins in ERMCSs**			
Cell fractionation		• Major technique to isolate the fraction of ERMCS and identify its protein components	• Purity is hard to guarantee
Ascorbate peroxidase (APEX) Tagging		• Identify new contact-site proteins	• Combining it with biochemical cell fractionation will reach a better purity	• Technically challenging	• Needs careful control

### Study of ERMCS Morphology and Structure

#### Electron Microscopy

Transmission electron microscopy (TEM) is still considered as the gold standard to study the structure of ERMCS. TEM analysis provides static, high-resolution, and ultrastructure information about the contact sites. Transmission electron microscopy is particularly suitable for samples with a large number of contact sites or those with resident proteins that can be analyzed by immune electron microscopy analysis (Honscher et al., 2014).

Electron tomography (ET) provides a three-dimensional view of the subcellular structures. In ET, a micrograph of a sample is recorded by tilting the sample in different directions and then merged into a three-dimensional structure. Electron tomography often requires serial sectioning, which could be laborious and technically challenging. Additionally, the incomplete 3D reconstructions with regions lacking information often leads to “missing wedge” artifacts (Fernández-Busnadiego, 2016).

Volume EM techniques based on scanning electron microscopy (SEM) not only provide high-resolution 3D images of large specimen volumes but also overcome the “missing wedge” artifacts (Peddie and Collinson, 2014). However, volume EM needs a powerful computer to process large datasets.

#### Epifluorescence and Confocal Microscopy

The most common technique to visualize the contacts is to express fluorescent proteins that label mitochondria and ER in the cells and observe the overlapping signals. This simple application is suitable for both static and live-cell imaging and is a readily available tool to study ERMCSs. However, this technique is associated with some inherent limitations because of its optical diffraction limit and some artifacts introduced by fixation (Scorrano et al., 2019). The development of super-resolution microscopy has enabled the high-resolution observation of ERMC dynamics (Guo et al., 2018). However, super-resolution microscopy does not conclusively provide information on the formation of contacts between two spatially close organelles.

A drug-inducible Forster resonance energy transfer (FRET)-based reporter was developed to visualize ERMCSs (Csordás et al., 2010). This technique involves introducing a rapamycin-induced dimerization domain, which can provide temporal quantitative measurements of contact distance. However, prolonged drug treatments can introduce artifacts. Several groups have reported the use of ERMCS reporters using split green fluorescent protein (GFP; Cieri et al., 2018; Yang et al., 2018). One half of the split GFP protein labels the ER surface, while the other labels the mitochondrial surface. The fluorescence of GFP is detected if the ER and mitochondria are spatially close enough to form contacts. The narrow (8–10 nm) probe and wide probe (10–50 nm) with short or long linker could be used to examine the narrow and wide contacts, respectively. These reporters do not artificially increase tethering between ER and mitochondria. Additionally, a light-inducible ER–mitochondria tethering (LIT) system was developed to temporally regulate the tethering between ER and mitochondria (Shi et al., 2018). This will be useful for studies that require multiple switching on–off steps and for avoiding side effects caused by continuous ER–mitochondria tethering.

#### Split Rluc8

*Renilla* luciferase (Rluc) is a bioluminescent enzyme. The reconstitution of split fragments of RLuc enables the detection of two closely associated proteins. Lim et al. (2015) developed a tool with Mito–RLuc8N and RLuc8C–ER to label the mitochondria and ER, respectively. When the ER and mitochondria are far apart, the activity of RLuc8 cannot be reconstructed. When the ER and mitochondria form contacts, Mito–RLuc8N will be close to RLuc8C-ER, and the total enzyme activity of RLuc8 is detected through the luminescence conversion of substrates (Lim et al., 2015).

#### Proximity Ligation Assay

Proximity ligation assay (PLA) is an antibody-based method for detecting biomolecules and their physical proximity at a single molecule level resolution. Typically, two unique proteins are recognized by primary antibodies raised in different species. The secondary antibodies conjugated with a pair of oligonucleotides (PLA probes) bind to the primary antibody. The PLA probes will hybridize with the connector oligos only if they are in close proximity to each other. The ligase forms a closed, circle DNA template, and the PLA probe acts as a primer to generate concatemeric sequences, which amplify the signals that are still tethered to the PLA probe by 1000-fold. The labeled oligos hybridize to the complementary sequences within the amplicon, which could then be analyzed by microscopy analysis. This technique was first introduced in 2002 to detect Zeptomoles (10 or 21 mol) of platelet-derived growth factor (PDGF) in the solution phase (Fredriksson et al., 2002). Currently, this assay is mainly used to detect the proximity between the two proteins (Bagchi et al., 2015). In PLA, two contact specific proteins on the ER side and mitochondria side are recognized by their antibodies, and the signals could quantitatively indicate the distance or contact range between the two membranes.

### Detection of Resident Proteins in ERMCSs

#### Cell Fractionation

Cell fractionation mainly refers to the separation of organelles by sucrose gradient. In 1957, Lever (1958) demonstrated that the mitochondria separated by sucrose gradient were always mixed with ER, which indicated the association between ER and mitochondria. Currently, cell fractionation is still a major technique to isolate the fraction of ERMCSs and identify its protein components. The purity of the fraction obtained remains a concern.

#### Ascorbate Peroxidase Tagging

*In situ* proximity labeling method uses an engineered ascorbate peroxidase (APEX) to tag a protein of interest and evaluate its expression. The cells are then treated with hydrogen peroxide (H_2_O_2_) in the presence of biotin–phenol. The proteins proximal to the APEX fusion protein will be covalently labeled with biotin, which could then be purified and detected via mass spectrometry. Using this APEX tagging technique, the ERMCS-specific protein could be tagged with APEX, and the other proteins located at the ERMCSs could be identified (Cho et al., 2017).

In conclusion, ERMCSs are hot spots for multiple cellular events and are critical for neuronal homeostasis. Although there is no direct evidence to indicate that changes in the ERMCSs can cause neurodegeneration, further studies on ERMCs will provide valuable insights into neurodegeneration and potentially a novel therapeutic target. As mentioned earlier, there are inconsistencies regarding some findings related to the roles of ERMCSs in neurodegenerative diseases. The rationales underlying these remain elusive. Developing a standardized approach to analyze and quantify ERMCSs might be the key to resolve the inconsistencies.

## Author Contributions

CT obtained financial support. All authors wrote and approved the final version of the manuscript.

## Conflict of Interest

The authors declare that the research was conducted in the absence of any commercial or financial relationships that could be construed as a potential conflict of interest.
